# New Meanings in the Archive: Privacy, Technological Change and the Status of Sources**

**DOI:** 10.1002/bewi.202200027

**Published:** 2022-09-09

**Authors:** Jenny Bangham

**Affiliations:** ^1^ School of History Queen Mary University of London

**Keywords:** Archives, invisibility, Wellcome, blood, human genetics, genomics.

## Abstract

This essay reflects on how technological changes in biomedicine can affect what archival sources are available for historical research. Historians and anthropologists have examined the ways in which old biomedical samples can be made to serve novel scientific purposes, such as when decades‐old frozen tissue specimens are analyzed using new genomic techniques. Those uses are also affected by shifting ethical regimes, which affect who can do what with old samples, or whether anything can be done with them at all. Archival collections are subject to similar dynamics, as institutional change and shifts in ethical guidelines and privacy laws affect which sources can be accessed and which are closed. I witnessed just such a change during my research into human genetics using archives in the Wellcome Collection. A few years into my project, those archives had their privacy conditions reassessed, and I saw how some sources previously seen as neutral were now understood to contain personal sensitive information. This paper describes the conditions of this shift—including the effects of technological change, new ethical considerations, and changing laws around privacy. I reflect on how these affected my understanding of the history of human genetics, and how I and others might narrate it.

Archival collections are dynamic: institutional changes or shifts in ethical guidelines and privacy laws can affect which sources can be accessed and which are closed. Archivists must achieve a balance between privacy and access, in contexts, far beyond the library, that are continually shifting over time.^1^ I witnessed such a shift during my PhD research into the relationship between mid‐twentieth‐century genetics and the practices of blood transfusion. The initial idea for my project had been indirectly seeded by the acquisitions team of the Wellcome Collection, who, in the late 1990s and early 2000s, amassed a formidable collection of papers on the history of British blood group research, papers that centered on the London‐based, MRC‐funded scientists Robert Race, Ruth Sanger, and Arthur Mourant. The three were closely associated with Britain's wartime and postwar transfusion services, and they had used that association to build up extensive programs to investigate the serological complexity, genetics, and population diversity of blood groups. They were keen correspondents, and their large and enormously rich archives were a treasure trove of information about how blood donors, and the bureaucracies designed to manage them, helped to create a science of human heredity.

I became particularly interested in the ways that Race and Sanger's research depended on their methods for singling out intriguing samples from blood donors and hospital patients, and how they struck up scientifically productive relationships with those donors and patients and their families, sometimes over many years. But although the archives provided valuable access to some otherwise obscure people and their activities, I often found it challenging to recover the lives, experiences, and motivations of people beyond the main protagonists. This was in part because invisibility was built into the transfusion infrastructure. Large‐scale extraction, storage and transportation were only possible because of the efficiencies yielded by standardization and routine.^2^ The female blood grouping technicians and clerks on whose labors the service depended were numerous and interchangeable with respect to the system that they served. The transfusion service kept careful track of the donors it recruited, but it mostly obscured their contributions as individuals owing to the sheer numbers of people who volunteered and massive volumes of blood it mobilized. Meanwhile, researchers took to labeling and marking the genetic diversity of contentious groupings of peoples based on (using historical terminologies) race, tribe, and nation, as well as geography and religion, which flattened donors into categories that eclipsed other forms of personal identity.

These were all invisibilities deliberately built into the system, so to speak. But mid‐way through my own project I came to realize that my understanding of this system was also affected by regulations concerning the privacy of the people represented in the archives. The politics of those sources affected what I could see of the institutions and politics of blood transfusion and genetics.^3^ This came to my attention when the archives I was using had their privacy conditions reassessed, and I saw how some sources were now understood to contain sensitive personal information. What had caused this shift?

## 1. Dynamic Archives

The Wellcome Collection acquired its blood group archives between 1998 and 2004.^4^ In 2010, a decade into the postgenomic era, the library decided to reframe those collections when it incorporated them into a program to make its materials relating to human genetics freely available online, a £3.9 million digitization effort that it dubbed “Codebreakers: Makers of Modern Genetics.”^5^ The incorporation of the blood‐group papers into “Codebreakers” represented a historiographical change. Before “Codebreakers,” the blood‐group papers were three of many valuable collections within the library's holdings; now the Wellcome positioned the blood group collections as foundational in the canon of human genetics, alongside the work of Rosalind Franklin, Sydney Brenner, James Watson, and many others.^6^ The wartime cultivation of the altruistic sharing of blood, freely given to benefit community and nation, could now be seen as foundational to human genetics.^7^ This new level of visibility of the blood group papers was further enhanced by their digitization, which made the collections free for anyone to view, subject to registration on the library's website.

The Codebreakers venture might be viewed alongside efforts by the Wellcome to make its buildings and collections (of objects, manuscripts, and books) more widely accessible to a broader range of public audiences. This vision was considered by a committee established by the Wellcome in 1996 to assess the future role and direction of its research institute and library. At the time, the library was used principally by historians of medicine, many of whom were associated with the academic Wellcome Institute for the History of Medicine (as it was then called) in the same building. One of the committee's conclusions was that the library “could be used more and in particular could appeal to a wider audience […] beyond the Library's core user group, historians of medicine.”^8^ Twenty‐five years later, the ambition appears to have been strikingly realized. The library's website now hosts a stunning array of digital images of its archival holdings, which online visitors can access after providing their name, proof of identity, and address. In‐person visitors to 183 Euston Road are greeted by a sweeping new staircase to multiple floors of exhibition spaces, two cafes, and a sumptuously renovated library that includes a large lavishly designed reading room with soft chairs and sofas, free for any library members to use. More recently, the Wellcome has aligned its efforts to open its collections with its response to critiques of its colonial legacies, which includes a commitment to making the history of those collections more transparent.^9^

Back on the cusp of the millennium, these efforts toward “accessibility” chimed with the Wellcome's promotion of the free “sharing” of genomic data.^10^ In 1996, the Wellcome was one of the key institutions to help establish the Human Genome Project's “Bermuda Principles,” whereby institutions involved in the sequencing of human genomic data were committed to publishing it online within 24 hours of its creation.^11^ These commitments also resonated with the Wellcome's highly public leadership of the “Open Access” publishing movement, which, starting in the early 2000s, sought to establish a new model of scientific publishing whereby researchers would pay to publish their work, so that readers could access it without cost.^12^ Quoted in the newspaper *The Guardian*, one Wellcome representative explained that scientific knowledge “should be freely available to anyone who wants to read it, for whatever purpose they need it.”^13^ All of these initiatives contribute to the image of the Wellcome as an institution committed to the modern liberal democratic values of inclusion and participatory governance.^14^

However, as human genetics and genomics expanded, interest intensified in the meaning of privacy in relation to personal genetic data.^15^ Indeed, the increasingly widespread sharing of genetic and genomic data in the late 1990s coincided with the UK's Data Protection Act of 1998, which put in place regulations designed to protect “personal data” stored on computers or in organized filing systems, especially “sensitive personal data,” which included information about a person's physical health or condition.^16^ In line with this new law, the Wellcome archivists felt they needed to reassess some of their collections, especially those that had been catalogued before the Data Protection Act.^17^ So as Wellcome archivists started the process of digitizing the “Codebreaker” papers, they also re‐evaluated the privacy conditions of the archives relating to blood groups.

This re‐evaluation process was labor‐intensive, involving several members of the Wellcome Archives and Manuscripts team. The scale of the archives was bigger than any that the team had dealt with before, and although it was technically unstructured, the archivists chose to handle it as though it were structured, that is, processed data, and therefore subject to the Data Protection Act. Their crucial task was to evaluate whether any one file among the blood‐group papers contained sensitive personal data.^18^ They flagged files with documents containing donor lists, full names, and blood groups, or family pedigrees. In some instances, the archivists found that a series of letters might be capable of attaching a blood group to a personal name, pedigree, or family. Ruth Sanger and Robert Race had built up relationships with those they tested, so the archivists perceived that they had to consider files in the context of the whole collection. Viewed in isolation, a series of letters might not be classed as sensitive, but that could change if the series was viewed alongside other documents. Depending on the proportion of sensitive documents in a file, that file might be classified as “restricted access,” meaning that I and other readers could consult the papers but not copy or quote them, while others (with a larger proportion of sensitive documents) might be “closed,” in some cases for several more decades.

This protected the identities of donors and patients who made some of the very earliest corporeal contributions to genetics and serology, potentially including those donors who consented for their names to be published in journals, and whose names became attached to blood groups.

The tightened protections around the identities and medical data of donors and patients do not just constitute a shift of a culture of privacy (although that is important). It also points to a change in what biomedical samples and data *are*, and what exchanges of such objects mean. In the 1940s and 1950s, disembodied human blood was a substance that could be tested for and marked with a handful of blood groups; it could be used for therapeutic transfusion, and it was sometimes used to make pedigrees or population datasets for research into inherited associations with other human traits. But in the intervening decades, blood has been transformed into a substance that potentially reveals precise details of a person's identity. In the twenty‐first century, genomic data has become subject to the powerful logics of informatic capitalism and its prolific markets for personal information.^19^ Both blood and data given in the 1940s and 1950s might now be tethered to data‐gathering practices that could affect a family's access to healthcare or insurance.

Recent historical and anthropological scholarship has examined the ways in which old biomedical samples can be made to serve novel scientific purposes, such as when decades‐old frozen tissue specimens are analyzed using new genomic techniques.^20^ They have also shown how the potential uses of biological specimens are affected by changing ethical regimes, which help to define who can do what with old samples, or whether anything can be done with them at all.^21^ My encounters with the Wellcome archives taught me that the new uses and meanings of blood that have been created since the 1950s had the potential to impact not only the uses of samples and specimens, but also the records and correspondence that document their history.

New meanings and classifications have changed (and sometimes restricted) what can be done with those records, and what can be understood about their history. These important changes put out of my reach sources that I had hoped would contain clues as to which individuals had chosen (or had been persuaded or coerced) to be part of blood‐related scientific and medical endeavors, why they participated, and how they cultivated their relationships with researchers.

## 2. Reflections

As Stephen Hilgartner writes, “What a letter in the archive or an interview transcript, means—what it even *is*—forever remains fundamentally subject to modification through change in science, in scholarship, and in wider worlds.”^22^ For the blood group archives in the Wellcome Collection, technological changes in genetics and genomics had altered the broader context in which the archives were maintained and given meaning, and as a consequence their custodians had had to restrike the “balance between privacy and access” (to return to the phrase I opened with). Several shifts had occurred in concert. Modern human genetics had become a field widely understood to offer powerful insights into our identities, history, and our health, and the Wellcome's librarians had chosen to reframe the history of blood group research by positioning it as foundational to that field. The power invested in genetics and genomics had also become an important motivation for making the resources pertaining to those more broadly accessible to more people. And as technological changes had expanded what could (potentially) be understood from blood and data, so privacy protections had rightly tightened around the blood and paper trails of postwar donors and patients. Hilgartner observes that all knowledge objects are constituted by control relationships in mind: when, e. g., data, archives, images, or journal articles are created, classified, and framed, they are always done so in relation to the audiences that will read and use them.^23^ And as we see with the Wellcome archives, those control relationships have shifted with changes in the way that information can be circulated and combined and utilized. The ongoing production of genomic knowledge and its meanings in public and political life also affects the continuing creation of historical knowledge.

The repositioning of blood groups as scientific objects that were central to the history of human genetics created new partial invisibilities in that historical record.^24^ As a novice historian working on my first project, I found those potential silences and invisibilities both intriguing and worrying. How was I to include in my account of the structural and political conditions of biomedicine, the people who labored to clean instruments, carried out routine testing, offered samples, or (knowingly or not) participated in experiments? How did my own prejudices, familiarities, or institutional settings affect what I can see? Scholars of colonial history have been particularly observant of the ways in which historical narratives are shaped by the (changing) politics of sources, by the agendas and responsibilities of the institutions that house them, and by the labors of archivists and librarians who attend to their ongoing meanings.^25^ Anthropologist and historian Michel‐Rolph Trouillot analyzed the operation of power in the creation of historical narratives, and drew attention the silences and invisibilities that are made at every step of their construction: in the making of sources; the assembly and classification of archives; the creation of narratives, and the making of history in the moment of retrospective significance.^26^ As Rheinberger himself explains, it is useful to be aware of the political, institutional, and practical conditions of our historical sources, as well as the science they describe.^27^ The Wellcome's blood group archives taught me that conditions of both projects are closely intertwined.

## Footnotes

n1 I am paraphrasing from Sillitoe 1998.

n2 On the (partial) invisibility of infrastructures, see Star and Ruhleder 1996.

n3 Rheinberger 1998 discusses how the “politics of sources” affects what historians can see and know about the past.

n4 The collection was received by the National Cataloguing Unit for the Archives of Contemporary Scientists, University of Bath, from the Société Jersiaise and Mrs. Jean Mourant. They were transferred to the Wellcome Library in 1999 (Acc. No. 816): Timothy E. Powell and Nicola Ashbridge, “Arthur Ernest Mourant FRS (1904–1994). List of Papers in The Wellcome Library for the History and Understanding of Medicine” (National Cataloguing Unit for the Archives of Contemporary Scientists, University of Bath, 1999), author's own collection.

n5 The ‘postgenomic era’ is a phrase used to denote genetic research after the publication of the human genome. The title of this digital collection presumably alluded to efforts to crack the genetic code; the press release: “Million‐Page Story of Modern Genetics Launched by the Wellcome Library,” 4 March 2013, online: https://wellcome.org/press‐release/million‐page‐story‐modern‐genetics‐launched‐wellcome‐library (accessed 23 Month 2022); for a Wellcome blog post: Simon Chaplin, “Codebreakers: Makers of Modern Genetics,” 1 March 2013, http://blog.wellcomelibrary.org/2013/03/codebreakers‐makers‐of‐modern‐genetics/ (accessed 28 October 2020).

n6
https://wellcome.org/press‐release/million‐page‐story‐modern‐genetics‐launched‐wellcome‐library

n7 For more on the geneticization of the clinic, see Ilana Löwy, this issue (accessed 23 March 2022).

n8 The committee undertook an 18‐month audit of the aims and procedures of the Wellcome Institute for the History of Medicine, and prompted the formation of a working party to consider how the library might best be promoted, the results of which are described in: Hilton and Gold 1998.

n9 “The Colonial Roots of Our Collections, and Our Response,” 4 June 2021, https://wellcomecollection.org/pages/YLnsihAAACEAfsuu (accessed 23 March 2022).

n10 I use quotation marks for “accessibility” and “sharing,” and later for “Open Access,” because these terms have the potential to obscure the politics of communication and exchange; see, e. g.: Hilgartner 2017; Reardon 2017.

n11 On the creation and consequences of the Bermuda Principles, see: Hilgartner 2017, chapter 6; Maxton Jones et al. 2018. On debates about whether such open data could be subject to patents and licensing, see Reardon 2017, chapter 2.

n12 See, e. g., *The Times* 2004. For more updated policies on Open Access: https://wellcome.org/grant‐funding/guidance/open‐access‐guidance/open‐access‐policy. The Wellcome established its own Open Access journal in 2012, *eLife*: https://elifesciences.org/.

n13 Jha 2012.

n14 On the genomic liberalism of the new millennium, and debates about privacy, data sharing, and their consequences for social justice, see Reardon 2017. For more on the question of who benefits from so‐called open science, see, e. g., Staunton et al. 2021.

n15 For a longer cultural historical account of privacy in Britain, see Cohen 2013.

n16 https://www.legislation.gov.uk/ukpga/1998/29/contents/enacted (accessed 2 June 2022). The act pertained to “structured” or “processed” data, and although the archives in question were technically unstructured, the archivists chose to handle them as though they were subject to the Act. Moreover, the Act had some exceptions for data to be used in “Research, history and statistics,” but these did not refer to “sensitive personal data,” which was also subject to specific forms of explicit consent.

n17 The Wellcome archivists routinely review all collections that contain potentially sensitive information. For details of this reevaluation process, I am grateful to Wellcome archivist Toni Hardy: private communication with author, 20 June 2016; 5 August 2016; 21 June 2022.

n18 The Data Protection Act did not specifically mention genetic data; rather it stipulated that “sensitive personal data” included information as to “the racial or ethnic origin of the data subject,” “his sexual life” or “his physical or mental health or condition,” all of which the archivists found relevant to their assessment of the blood group archives: https://www.legislation.gov.uk/ukpga/1998/29/section/2/enacted (accessed 15 July 2022).

n19 On the landscape of twenty‐first‐century genomic capitalism, see Reardon 2017.

n20 Radin 2017.

n21 Kowal 2013; Radin and Kowal 2015.

n22 Stephen Hilgartner, this issue.

n23 Stephen Hilgartner made these observations during the discussion of the workshop “History & Historiography of the Life Sciences: Traces of Hans‐Jörg Rheinberger,” Berlin, 7–8 April 2022. See also Hilgartner 2017, on 7.

n24 For more on invisibilities in science and its histories, see Bangham et al. 2022.

n25 Among many rich analyses: Habib 2008; Stoler 2010; Roque and Wagner 2012; Caswell 2014.

n26 Trouillot 1995, on 26.

n27 Rheinberger 2008.
